# How can we augment the few that remain? Using stable population dynamics to aid reintroduction planning of an iteroparous species

**DOI:** 10.7717/peerj.6873

**Published:** 2019-05-02

**Authors:** Brenda J. Hanley, Elizabeth M. Bunting, Krysten L. Schuler

**Affiliations:** Cornell Wildlife Health Lab, Department of Population Medicine and Diagnostic Sciences, Cornell University, Ithaca, NY, United States of America

**Keywords:** Asymptotic dynamics, Resource management, Population dynamics, Eigenvalue, Recovery, Transient dynamics, Stable stage distribution, Stable stage proportions, Interactive software, Reintroduction planning

## Abstract

Restoration of depleted populations is an important method in biological conservation. Reintroduction strategies frequently aim to restore stable, increasing, self-sustaining populations. Knowledge of asymptotic system dynamics may provide advantage in selecting reintroduction strategies. We introduce interactive software that is designed to identify strategies for release of females that are immediately aligned with stable population dynamics from species represented by 2-, 3-, 4-, and 5-stage life history strategies. The software allows managers to input a matrix of interest, the desired number of breeding females, and the desired management timeline, and calls upon stable population theory to give release strategies that are in concert with both stable population status and the management goals. We demonstrate how the software can aid in assessing various strategies ahead of a hypothetical restoration. For the purpose of demonstration of the tool only, we use published vital rates of an ungulate species, but remark that the selection of species for demonstration is not central to the use of this tool. Adaption of this tool to real-life restorations of any 2-, 3-, 4-, or 5-stage iteroparous species may aid in understanding how to minimize undesirable recovery complications that may naturally arise from transient population dynamics. The software is freely available at: https//cwhl.vet.cornell.edu/tools/stapopd.

## Introduction

Global biodiversity loss has prompted diverse efforts to stem or reverse declines for many species (Kissel et al., 2014). Restoration plans regularly aim to recover critically low populations to some predetermined level (Wiedenmann, Fujiwara & Mangel, 2009). We introduce software that generates reintroduction strategies that are immediately aligned with long-established equations of stable population theory. The software written in R programming language (RStudio Team, 2015; R Core Team, 2018; R Shiny, 2018), and is designed to aid in planning ahead of restorative reintroductions of iteroparous floral or faunal species with 2-, 3-, 4-, or 5-stages in their life history.

Calculations involving the dominant eigenvalues (“asymptotic growth rate”) of stable population theory are regularly utilized in population viability analyses (PVA). Among other uses, PVA constitute an important class of analyses in developing reintroduction protocols (Morris & Doak, 2002; Pacioni & Mayer, 2017). Standard methods for PVA include population-based models (Starfield & Bleloch, 1986; Burgman, Ferson & Akcakaya, 1993), which are extensions of traditional methods of population ecology and demography (Lacy, 2000). Alternatively, individual-based models are a type of PVA that simulates the life history events of individuals in the population, and then monitors the status of each individual and the population as a whole (Lacy, 2000). Herein, we advance the use of population-based models in reintroduction planning and suggest that individual- and population-based models may be considered in parallel in reintroduction planning.

The population matrix model (PMM) uses stable population theory to link the vital rates to the asymptotic system dynamics that they generate (Tuljapurkar, 1997; Caswell, 2001). The PMM is structured into ages or stages according to the life history of the model organism (ages; Leslie matrix, Leslie, 1945; stages; Lefkovitch matrix, Lefkovitch, 1965; herein we generalize the terminology to “stage”). The matrix elements represent the biological transitions within the modeled life history (see Caswell, 2001).

The PMM has been used to calculate the asymptotic growth rate of a population (Caswell, 2001). As well, it has been used to investigate how modifications to either matrix elements or stage abundances incite differing system dynamics (Tuljapurkar, 1997; Caswell, 2001). Techniques to understand such differential impacts have resulted in extensive attention on sensitivity analyses (De Kroon, Van Groenendael & Ehrlen, 2000; Caswell, 2001). Such inventory and sensitivity analysis capabilities have been used to enhance understanding of how vital rates influence population trajectories of nearly 8,000 floral and faunal systems across the globe (Salguero-Gómez et al., 2014; Salguero-Gómez et al., 2016).

The PMM describes two types of system-level dynamics; ‘asymptotic’ (stable) dynamics, and ‘transient’ (non-stable) dynamics (Fox & Gurevitch, 2000; Wiedenmann, Fujiwara & Mangel, 2009; McDonald et al., 2016). The “asymptotic quantities” include the dominant eigenvalue (“growth rate”), subdominant eigenvalues, damping ratio, sensitivities, and elasticities (see Caswell, 2001). Stable dynamics occur when the change in stage abundances across time are influenced by the dominant eigenvalue only (Caswell, 2001). The stable stage distribution (SSD; SSP is the abbreviation for stable stage proportions) constitutes the abundances of individuals that must exist in each stage for the system to exhibit stable dynamics (Caswell, 2001). The system-level “transient” (non-stable) dynamics include attenuation, amplification, inertia, and momentum (see Ezard et al., 2010). Non-stable dynamics occur when stage abundances deviate from SSD, or equivalently, when the changes in stage abundances across time are influenced by all eigenvalues, not just by the dominant (Fox & Gurevitch, 2000). Non-stable populations exhibit non-patterned dynamics, where size, growth, and structure exhibit behavior that are dissimilar to stable dynamics (Stott, Hodgson & Townley, 2012; Stott, 2016).

A frequent goal of reintroduction is to produce a stable, growing, self-sustaining population. Although transient dynamics may in some cases be beneficial to introductions (Stott, Hodgson & Townley, 2010), we demonstrate that in other cases transient dynamics may not be desirable from the managerial perspective of restoration. The software allows users to compare stable and non-stable trajectories in the context of their systems and gives suggestions for reintroduction strategies that are immediately aligned with stable population dynamics. The software enables managers to assess whether stable or transient dynamics are more desirable in the situational context of their restorations. The software is available at: https//cwhl.vet.cornell.edu/tools/stapopd.

## Materials & Methods

Let **L** be an arbitrary *m*-stage primitive PMM that represents a single sex (female) of a density-independent species that is operating in a closed system. Let *T* represent discrete time units and let ***n***_*T*_ be the *m* × 1 vector of female stage abundances at time *T*. Assuming a static (deterministic) **L**, population abundances of females can be projected *T* time units (Caswell, 2001):

(1) \begin{eqnarray*}& & {\mathbi{n}}_{T}={\mathbf{L}}^{T}{\mathbi{n}}_{0}.\end{eqnarray*}

Initially Koons, Holmes & Grand (2007) and then later Kissel et al. (2014) suggested that managers should consider releasing individuals in their stable stage distribution (SSD). For the purposes of this derivation, we therefore assume that releases are made in SSD, that is, that ***n***_0_ is in SSD. Please see the supplement for equations regarding the calculation of SSD. We further assume that target restoration goals have been predetermined; the proposed timeframe (*T*) and the desired number of breeding females at time ***n***_*T*_ are known. The release abundances necessary to achieve the predetermined population goal in a manner aligned with stable population theory is then:

(2) \begin{eqnarray*}& & {\mathbi{n}}_{0}= \frac{1}{{\lambda }_{1}^{T}} {\mathbi{n}}_{T},\end{eqnarray*}

where *λ*_1_ is the asymptotic growth rate of the population. We highlight that both ***n***_0_ and ***n***_*T*_ must be in SSD for Eq. (2) to logically follow Eq. (1). The full derivation appears in the supplement.

The interactive software uses Eq. (2) in conjunction with predetermined user inputs (“My matrix requirements”, “Desired management goals”) to produce several restoration strategies (“Candidate models”) that are aligned with stable-status (“Stable proportions”, “Target abundances at time zero”, “Target abundances at time one”), or to see what would happen should a release occur out of stable-status (“Reintroduction out of SSD).

“My matrix requirements” tab. This tab allows users to enter the magnitude of each element in their PMM of interest. The layout of the matrix in the app is identical to traditional layout of PPMs (e.g., Caswell, 2001); the columns represent the stages, in ascending order with the *i*th stage represented by the *i*th column. The entry boxes in the top row represent the fertilities (or fecundities) of each stage. All remaining elements are considered “transitions”. A transition in the *i*th diagonal represents the average probability that a female in the *i*th stage will survive a time unit and remain in the *i*th stage. A transition in the *i*th off-diagonal represents the average probability that a female in the *i*th stage will survive a time unit and simultaneously transition out of the *i*th stage. The units of the fertilities are the average female offspring per breeding female per time unit. Depending on the species, fertilities at certain stages may be negligible or biologically implausible. The fertilities therefore may take on the value of 0 (for no reproduction of that stage) or any positive decimal. Similarly, transitions may take on decimal values between 0 and 1. The software user may obtain help by clicking on the “Help me determine what to enter” button. As well, background information regarding the population matrix model is obtained by clicking the “Foundational matrix model information” button. After each matrix element is entered by the user in the appropriate entry box, a click of the “Save matrix entries” button will activate the “Desired management goals” tab.

“Desired management goals” tab. This tab allows users to enter the desired management information of their reintroduction; desired number of time units (*T*), desired abundances of breeding females at time *T*, and the desired number of candidate models to compare. The user may obtain help by clicking on the “Help me determine what to enter” button. After each management goal is entered in the appropriate entry box, a click of the “Calculate candidate models” button will activate the “Candidate models” tab.

“Candidate models” tab. An array of numbers will automatically appear in the body of this tab. Each row of the array contains information from a single matrix model, unraveled row-wise and reformatted into a single row. The first row in the array contains the vital rates of the PMM previously entered by the user, the corresponding finite rate of growth (“dominant eigenvalue”) for that PMM, and the total number of females (in all stages) that need to be released at time *T* = 0 to propel trajectories toward the target management goals. Rows 2 through *x* of the array (where *x* is a number specified by the user) includes models with similar life history structure to the PMM entered by the user. Drawing from a pre-saved data set containing thousands of simulated PMMs which themselves contain randomly generated elements, the software selects *x* − 1 pre-saved models that contain identical life history structure and similar vital rate magnitudes (+∕ − 0.5) to those entered by the user. The corresponding growth rates and release abundances of each *x* − 1 PPMs also appear in each row. The *x* − 1 pre-saved models are sorted by total release abundances and presented in ascending order in rows 2 − *x*, so that the most “efficient” alternative PPM (from the perspective of minimal release abundances) is listed directly below the user-entered model. The user may obtain help by clicking the “What does this mean” button. The user enters the row number of the model of interest, and a subsequent click of the “Calculate detailed information for this model” button will activate all the remaining tabs of the software interface.

“Selected population matrix model” tab. Selection of this tab reveals the model of interest in familiar matrix model form. Help is obtained by clicking on the “What is happening here?” button. The matrix will automatically update should the user decide to return to the “Candidate models” tab and select a different model.

“Stable stage proportions of the selected model” tab. Selection of this tab will reveal the stable stage proportions (SSP; SSD standardized to 1) of the selected model. The SSP is displayed in both pie chart and table formats. Help is obtained by clicking on the “How do I interpret this?” button. These stable-proportions hinge on the specific elements in the underlying model matrix and therefore will automatically update should the user decide to return to the “Candidate models” tab and select a different model.

“Target abundances in time zero for the selected model” tab. This tab displays the female abundances that must be in each stage at the onset of the restoration (*T* = 0) for the release to be in stable-status and aligned toward the target goals. Help is obtained by clicking on the “What do these numbers mean?” button. These abundances hinge on the specific elements in underlying model matrix in conjunction with the desired management goals, so the results in this tab will automatically update should the user return to the “Candidate models” tab and select a different model or return to the “Desired management goals” tab and update their desires.

“Target abundances though time for the selected model” tab. This tab displays the female abundances necessary in each stage in each time unit from the onset of restoration up to and including the end of the restoration timeline (*T* = *T*). Help is obtained by clicking on the “What do these numbers mean?” button. These abundances hinge on the specific elements in underlying model matrix in conjunction with the desired management goals, so the results in this tab will automatically update should the user return to the “Candidate models” tab and select a different model or return to the “Desired management goals” tab and update their desires.

“Reintroduction out of SSD” tab. This tab displays a graphical representation of population trajectories when release occurs in and out of stable-status. The tab automatically produces a plot of resulting trajectories in SSD. The user may enter any set of initial abundances so that situational comparison of trajectories in and out of SSD may be made. After all stage-wise release abundances are entered in the appropriate entry box, the display will automatically plot transient trajectories atop the stable trajectories. All trajectories hinge on the underlying model matrix in conjunction with the desired management goals, and so the plots will automatically update should the user return to the “Candidate models” tab and select a different model, return to the “Desired management goals” tab and update their desires, or create different sets of initial conditions.

We illustrate the capability of this software for a hypothetical restoration of an ungulate species using known model structure and vital rates as published in Chitwood et al. (2015). We demonstrate the use of the tool in a hypothetical restoration scenario where managers wish to grow a population from 0 to 100 breeding females in 7 years’ time. In the “My matrix requirements” tab, we enter the matrix entries for the PMM that describes the ungulate species (Chitwood et al., 2015):

\begin{eqnarray*}\mathbf{A}= \left[ \begin{array}{@{}ccc@{}} \displaystyle 0&\displaystyle 0.581&\displaystyle 0.701\\ \displaystyle 0.141&\displaystyle 0&\displaystyle 0\\ \displaystyle 0&\displaystyle 0.775&\displaystyle 0.801 \end{array} \right] . \end{eqnarray*}

On the “Desired management goals” tab, we enter “7” in entry box that records the desired restoration timeline, “100” in the entry box that records the desired female abundances, and “20” in the entry box that records the desired number of candidate models.

## Results

The software shows that 335 total female individuals must be released at *T* = 0 to align a restoration with stable population dynamics to theoretically produce 100 breeding females in 7 years’ time. The SSP contains ∼43% of stage-1 females, ∼6% of stage-2 females, and ∼50% of stage-3 females. Of the 335 released females at *T* = 0, 145 are stage-1, 18 are stage-2, and 172 are stage-3. Time *T* = 7 will theoretically contain 75 stage-1 females, 11 stage-2 breeding females, and 87 stage-3 breeding females. The total 100 desired breeding females is partitioned (in the proportion of SSD) between stages 2 and 3.

## Discussion

This demonstration indeed aligns the reintroduction with stable-status, however, there is a problem with this hypothetical plan! These example vital rates do not naturally produce an increasing population, so merely releasing in SSD will not necessarily produce a growth population, nor achieve the desired restoration objectives. Additional capabilities of the software can be used to align a restoration towards goals after all.

Increasing populations. An eigenvalue (“growth rate”) greater than 1 means that the population will increase, an eigenvalue less than one means the population will decrease, and an eigenvalue equal to 1 means that the population will remain the same. In the “Candidate models” tab, the eigenvalue for the hypothetical ungulate example is 0.904, indicating that the population would naturally decline by 10% per time unit. Indeed, the plot on the “Reintroduction out of SSD” tab illustrates a stable decline! Assessing population trends for eigenvalue magnitudes is a form of PVA. Managers who instead wish to produce stable growth must return to the “Desired management goals” tab and input different vital rates. This modification of vital rates can be thought of as a type of investigative sensitivity analysis.

Increasing populations; option 1. The “Candidate models” tabs shows several previously stored PMMs that are similar in structure and vital rate magnitudes to the PMM defined by the user. The second row shows the alternative model that requires the fewest release abundances. In the context of the ungulate example, row 2 reveals that managers could theoretically achieve their management goals by releasing 29 total females, provided managerial intervention is capable of increasing stage-2 and stage-3 fertilities to .93 and 1.12 (respectively), and capable of increasing stage-1 and stage-2 survival to .47 and .88 (respectively). Fertilities may be limited by biological reasons (and therefore cannot be manipulated by management), and so this “optimal” model may not be achievable. However, models that call for increased survival probabilities only may indeed be achievable though targeted management. After all, managers regularly influence survival rates of wildlife species in myriad ways. Software users may peruse the remaining *x* − 1 models and interpret the plausibility of the survival requirements required in each alternative model in the context of their reintroduction scenario.

Increasing populations; option 2. Managers may return to the “My matrix requirements” tab and may modify a single vital rate (or group of vital rates) to assess the reintroduction strategies for the updated model. For example, suppose it is possible for managers to increase survival of stage-3 in our example organism to:

\begin{eqnarray*}& & \mathbf{A}= \left[ \begin{array}{@{}ccc@{}} \displaystyle 0&\displaystyle 0.581&\displaystyle 0.701\\ \displaystyle 0.141&\displaystyle 0&\displaystyle 0\\ \displaystyle 0&\displaystyle 0.775&\displaystyle 0.95 \end{array} \right] . \end{eqnarray*}

The “Candidate models tab” shows that after this targeted modification to stage-3 survival, the resulting population will naturally grow at 2.8% per time unit. Furthermore, provided managers can maintain stage-3 survival at 0.95 over the 7 year time period, then 125 total females (stage-1: 53, ∼40%, stage-2: 2, ∼5%, and stage-3: 70, ∼54%) must be released at *T* = 0 to prime the system to grow in stable-status to theoretically reach 100 breeding females in 7 years’ time.

The software may reveal a disparity between theoretical stable abundances and pragmatic release abundances. Suppose the theoretical results call for far too many released females than feasible in reality. For example, managers may be faced with limited funding with which to captive breed or live trap animals, managers may not have the requisite time to grow or gather the requisite numbers, or the requisite abundances constitute more individuals than the remaining abundances left on the planet. Because a holistic approach to decision making also includes the financial realities of recovery (Kissel et al., 2014), the magnitude of theoretical release abundances may help managers assess the feasibility of stable-status reintroduction. The software provides a way to investigate two options; (1) see what would happen (in a deterministic sense) if managers were to release the abundances at hand (presumably out of SSD), or (2) see what would happen (in the same deterministic sense) if managers were to release fewer females yet maintain SSD proportions. The desirability of either option in practice is situational, and therefore the software only supplies illustrative trajectories (and not a decision rule).

**Figure 1:**
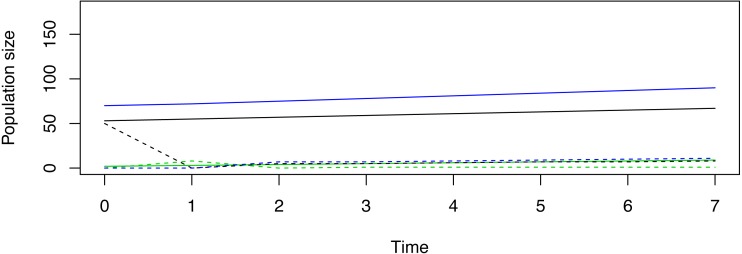
An example of a non-stable trajectory that arises when only stage-1 females are released. Deterministic trajectories for reintroduction in and out of stable status for a demonstrative species given the hypothetical goal to achieve 100 breeding females in 7 years’ time when only stage-1 females are released. The solid lines represent stage abundances released in SSD, while the dotted lines represent stage abundances when 50 stage-1 females are released without simultaneous release of females in stages 2 and 3. In one unit of time, the majority of the released stage-1 females have died. This immediate attenuation equates to a nearly comprehensive loss of the captive breeding (or live trapping) resources that were gathered ahead of the reintroduction. By the end of time 7, this reintroduction strategy has produced deterministic dynamics that are nowhere near achieving the restoration goal. This release strategy may not be desirable.

**Figure 2:**
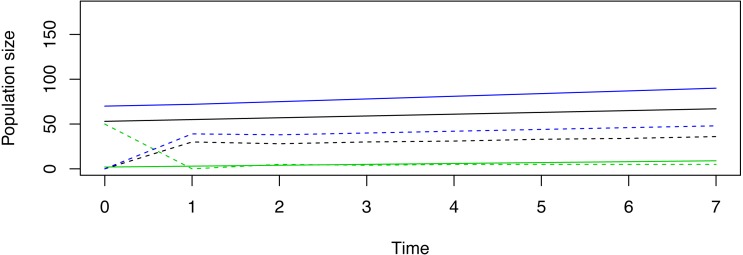
An example of a non-stable trajectory that arises when only stage-2 females are released. Deterministic trajectories for reintroduction in and out of stable status (SSD) for a demonstrative species given the hypothetical goal to achieve 100 breeding females in 7 years’ time when only stage-2 females are released. The solid lines represent stage abundances released in SSD, while the dotted lines represent stage abundances when 50 stage-2 females are released in isolation. Although this trajectory also experiences attenuation, because the released females both breed and transition to adults, their presence at release contribute new stage-1 and stage-3 females in the subsequent time unit. By the end of time 7, this reintroduction is closer to meeting the management goal than the strategy in Fig. 1.

**Figure 3:**
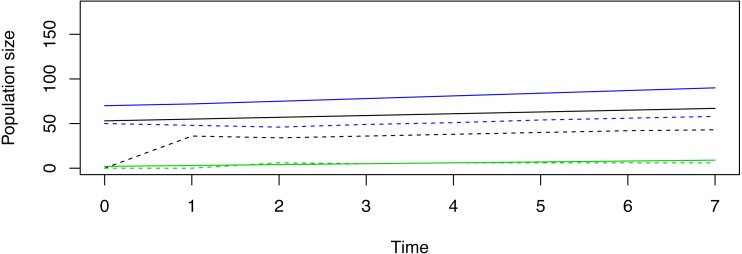
An example of a non-stable trajectory that arises when only stage-3 females are released. Deterministic trajectories for reintroduction in and out of stable status (SSD) for a demonstrative species given the hypothetical goal to achieve 100 breeding females in 7 years’ time when only stage-3 females are released. In this life history, this strategy of reintroduction is closer to meeting the management goal than the situations in Figs. 1 and 2. The efficacy of each situational strategy depends on the life history of the model organism coupled with the desired restorative goals.

Pragmatic constraints option 1. Suppose in our hypothetical demonstration that managers can release only 50 total female deer, and suppose the decision is made to release individuals that belong to only one stage. The “Release out of SSD” tab reveals that a release of 50 stage-1 females, 0 stage-2 females, and 0 stage-3 females might cause the reintroduction to immediately fail (Fig. 1). Alternatively, release of 0 stage-1 females, 50 stage-2 females, and 0 stage-3 females would initiate limited population fluctuations, but trajectories may be deemed to be more desirable (Fig. 2). Release of 0 stage-1 females, 0 stage-2 females, and 50 stage-3 females would also fail to achieve the management goal in the pre-specified timeline, but this release appears to induce the least amount of transient volatility when compared to the other two strategies (Fig. 3). Given this information, managers must decide which type of trajectory is most desirable.

**Figure 4:**
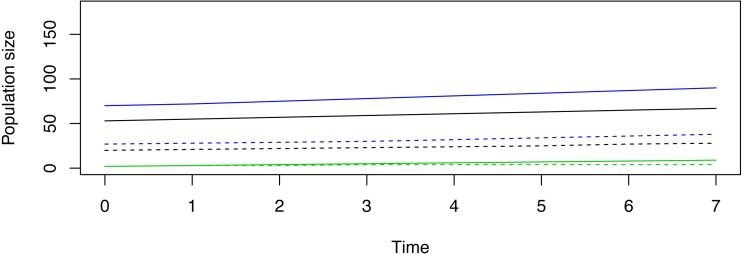
The scaling of an example reintroduction according to SSD. Deterministic trajectories for reintroduction in stable status (SSD) for a demonstrative species given the hypothetical goal to achieve 100 breeding females in 7 years’ time. The solid lines represent the stable trajectories that align with the target goal. The dotted lines represent stage abundances when only 50 total females are available to be released in SSD. By scaling down the abundances (but maintaining stable-proportions), the constrained reintroduction will take longer to achieve management goals, but the reintroduction itself is still aligned with stable growth.

Pragmatic constraints option 2. Suppose managers can release only 50 total female deer and opt to release in SSD. A release of 20 stage-1 females, 2 stage-2 females, and 27 stage-3 females (a total of ∼50 females) will yield trajectories that lengthen the time to achieve the management goal but maintain stable-growth (Fig. 4). Indeed, any “scaling up” or “scaling down” of release abundances in SSD will produce stable-trajectories.

While stochastic, individual-based models have seen a large uptake by managers and researchers due to their capacity to include (environmental) stochasticity and to quite accurately describe complex dynamics (Lacy, 2000), we show that comparatively simple population-based deterministic models may also be useful in investigating the pros and cons of restoration strategies (e.g., Starfield, 1997). Indeed, modeling a large diversity of population processes will lead practitioners to consider threats to population viability that would otherwise have been neglected (Lacy, 2000). Herein, we use the mathematics of the deterministic PMM to help managers reveal situational risks to restoration success that might have otherwise been overlooked.

The software uses stable population theory to aid in decision making from a variety of important perspectives. The “Target abundances in time zero” tab allows managers to prepare a suggested level of captive breeding (or live trapping) necessary for a stable-status release. The “Target abundances through time” tab allows managers to assess the trade-off between captive breeding for initial release and head-starting for later augmentation (e.g., Kissel et al., 2014). The “Stable proportions” tab allows managers to “scale up” or “scale down” a reintroduction effort while maintaining stable-status, and therefore extends the results to larger- or smaller-scale restorations. The “Reintroduction out of SSD” tab shows what deterministic population would do if managers release abundances that are limited by pragmatic constraints, brought about through opportunistic mechanisms, or out of convenience. Finally, the “Candidate models” tab, in conjunction with the capacity to change matrix elements, provides managers a way to investigate how a change in a matrix element will impact trajectories, release abundances, or stable-dynamics; all a contextual variation of sensitivity analysis.

Managers that are planning reintroduction may benefit from a tool that shows how stable, deterministic population dynamics may constitute a latent risk to restoration success. Stage abundances that are out of SSD will produce non-stable, non-patterned transient dynamics (Fox & Gurevitch, 2000) and may contain attenuations that heighten underlying risks of extinction (Wiedenmann, Fujiwara & Mangel, 2009; Stott, Hodgson & Townley, 2012). By investigating deterministic trajectories in and out of SSD, managers can ensure that their situational contribution to the population is aligned with—and not inadvertently antagonistic to—their restorative goals.

This software should not be used in isolation for restoration planning; it is crucial that numerous other ecological considerations must be considered in any restoration plan. For example, population abundances measured in individuals and the standing genetic variability measured in heterozygosity constitute differing measurements of population viability (Gotelli, 2004; Futuyma, 2009). Releasing individuals in SSD does not guarantee sufficient genetic variability to support a sustainable population. We suggest that management targets should aim to be established in consideration of both demographic abundances and genetic desired outcomes.

The assumption of high-density independence for this reintroduction software makes little impact on near term trajectories, provided no animals already exist in the recipient site. Since both the exponential growth curve and the logistic growth curve are of similar shape near zero, at the onset of a reintroduction, the curves do not differ, even if high-density dependence dynamics are known to exist in the species at larger abundances. However, the same cannot be said for low-density dynamics. We recommend that managers investigate impacts of Allee effects on their species prior to using this software to plan reintroductions. If this software is used to plan an augmentation of the current population in a recipient area, it is prudent to access all density dependent dynamics when planning restorations.

This model does not consider abundances of males, and therefore makes the post hoc extrapolation to total abundances critical. For example, for a sexually reproducing species with sex ratio of 1:1, the total number of released individuals at each stage at *T* = 0 must be doubled! It immediately follows that carrying capacity of the reintroduction site must always be taken into consideration. We recommend the use of this software only if the reintroduction habitat can support the total desired abundances.

The vital rates of **L** are assumed to be stage averages, and further, it is assumed that these averages remain static between *T* = 0 and *T* = *T*. Maintaining the habitat according to **L** might be easy in theory but unrealistic in practice. Unforeseen population perturbations will cause **L** to change, which will in turn invalidate deterministic projections. Since PMMs are known to be a sound way to quantify population status within the period of data collection (Crone et al., 2012), annual demographic surveys may be used to make interim adjustments in stage abundances that have arisen due to changes to **L**. Alternatively, should **L** change after reintroduction, investigations into transient dynamics may be necessary (see Koons et al., 2005; Koons, Grand & Arnold, 2006; Koons, Rockwell & Grand, 2006; Gerber & Kendall, 2016). It is prudent to have a contingency plan to assess the success of the restoration when **L** cannot be held (or assumed to be held) constant.

We assume that deterministic processes accurately reflect population-scale dynamics during time *T* = 0 to *T* = *T*. We acknowledge that several complications exist that may undermine the validity of our simplifying assumptions. For example, interactions between species at the reintroduction site may modify survival of the target population in ways that this model does not quantify. As well, issues with site fidelity may undermine the assumption of a closed system and may therefore modify stable-dynamics at the release site. This deterministic model also does not quantify how population-scale dynamics may differ as a result of imperfect sampling, demographic, or stochastic variation. It is prudent to assess each of these important considerations prior finalizing any reintroduction plan.

Despite these limitations, it is possible to improve the quantitative basis for decisions regarding alternative recovery actions when basic demographic data is available (Kissel et al., 2014).

## Conclusions

Reintroduction are complex, and this software does not replace careful, detailed planning on many relevant biological, environmental, social, or economic topics. However, developing tools to evaluate conservation strategies to decrease extinction risk are critical for imperiled populations (Kissel et al., 2014). This software offers strategies to aid in restorative planning from the isolated perspective of stable population theory. The software can be used in unison with individual-based models to better understand the holistic complexity of any biological restoration.

##  Supplemental Information

10.7717/peerj.6873/supp-1 Supplemental Information 1 Supplemental Derivations The mathematical derivations that underlie the software application.

10.7717/peerj.6873/supp-2 Supplemental Information 2 Zip folder of software The .txt files and R code that underlies the interactive software.

## Additional Information and Declarations

### Data Availability

The following information was supplied regarding data availability: This endeavor did not use any real data that was not already in the public domain. We did, however, generate and use simulated data. The .txt files that contain the simulated data, the raw R code that was used to simulate the .txt file data, and the raw R code that is used to run the interactive software application are found at: https://cwhl.vet.cornell.edu/tools/staPOPd. The StaPOPd software R code (and simulated data sets) are also available in the Cornell library eCommons system: https://doi.org/10.7298/d9hg-wa84.
