# Supplementary material for: How can we augment the few that remain? Using stable population dynamics to aid reintroduction planning of an iteroparous species
Source: PeerJ. 2019 May 2;7:e6873. doi: 10.7717/peerj.6873 (PMC6500717; doi:10.7717/peerj.6873)
Supplement: Supplemental Information 1 — The mathematical derivations that underlie the software application. [file peerj-07-6873-s001.docx]

Hanley BJ, Bunting EM, Schuler KL. 201X. How can we augment the few that remain? Using stable population dynamics to aid reintroduction planning of an iteroparous species.

SUPPLEMENTAL INFORMATION

Let $\mathbf{L}$ be an arbitrary $m$-stage primitive population matrix model:

$\mathbf{L}=\left[ \begin{matrix} a_{11} & \cdots& a_{1m} \\ \vdots& \ddots& \vdots\\ a_{m1} & \cdots& a_{mm} \end{matrix} \right]$, (1)

where $a_{1i}$ represents the fertility of the $ith$ stage, and $a_{2.}\ldots a_{m.}$ represent the biological transitions amongst age (or stage) classes. The fertilities may take on any positive value, while the transitions are constrained between 0 and 1. Let $t=1, 2, \ldots$ represent discrete time units, $\boldsymbol{n}_{0}$ be the (known) $m\times1$vector of stage abundances at time $0$, and $\boldsymbol{n}_{T}$ be the (unknown) $m\times1$vector of stage abundances at time $T$.

Matrix models utilize the information contained in the life history structure and vital rates ($\mathbf{L}$) with stage abundances ($\boldsymbol{n}_{0}$) to project how abundances will change in the future ($\boldsymbol{n}_{1}$). Population abundances at $T=1$may be calculated using the recursive equation (Caswell 2001):

$\boldsymbol{n}_{1}=\mathbf{L}\boldsymbol{n}_{0}$. (2)

Repetition of the process will yield population abundances after an additional time unit:

$\boldsymbol{n}_{2}=\mathbf{L}\boldsymbol{n}_{1}=\mathbf{L}\left( \mathbf{L}\boldsymbol{n}_{0} \right)=\left( \mathbf{LL} \right)\boldsymbol{n}_{0}=\mathbf{L}^{2}\boldsymbol{n}_{0}$. (3)

Following this pattern, population abundances can be projected $z$ time units into the future:

$\boldsymbol{n}_{z}=\mathbf{L}^{z}\boldsymbol{n}_{0}$. (4)

The total number of females at time $T$ ($N_{T}$) is obtained by adding abundances across all stages ($\boldsymbol{n}_{T}$) at time $T$.

Spectral decomposition of the model matrix transforms eq. 4 into:

$\boldsymbol{n}_{z}=\boldsymbol{v}_{1}\boldsymbol{n}_{0}\boldsymbol{\lambda}_{1}^{z}\boldsymbol{w}_{1}$+$\boldsymbol{v}_{2}\boldsymbol{n}_{0}\boldsymbol{\lambda}_{2}^{z}\boldsymbol{w}_{2}+\boldsymbol{v}_{3}\boldsymbol{n}_{0}\boldsymbol{\lambda}_{3}^{z}\boldsymbol{w}_{3}+\ldots+\boldsymbol{v}_{m}\boldsymbol{n}_{0}\boldsymbol{\lambda}_{m}^{z}\boldsymbol{w}_{m}$, (5)

where $\boldsymbol{w}_{i}$ represents the $ith$ right eigenvector associated with$ith$ eigenvalue ($\boldsymbol{\lambda}_{i}$), and $\boldsymbol{v}_{i}$ represents the inverse of $\boldsymbol{w}_{i}$ (Fox & Gurevitch 2000).

A frequent goal of the reintroduction is to introduce a population in a manner that eventually achieves a self-sustaining, growing, stable population. Individual animals that are used for reintroduction are typically obtained from captive breeding facilities, rehabilitation facilities, or from other wild-source populations (Kissel et al. 2014). Given that managers can control these initial conditions, we derive a model that assumes complete control over the abundances and proportions of animals at release. We select initial conditions that are precisely aligned with the conditions at the onset of the long-term, asymptotic behavior. In eq. 5, as $z$ goes to some critical time unit $c$, all subdominant eigenvalues tend to zero, leaving:

$\mathbf{n}_{c+1}=\mathbf{v}_{1}\mathbf{n}_{0}\boldsymbol{\lambda}_{1}^{c}\mathbf{w}_{1}$. (6)

We shift the time unit of interest for the reintroduction to $T=c=0$, leaving:

$\boldsymbol{n}_{1}=\boldsymbol{v}_{1}\boldsymbol{n}_{0}\boldsymbol{\lambda}_{1}^{1}\boldsymbol{w}_{1}$ (7)

We introduce a crucial assumption to this time shift that $\boldsymbol{n}_{0}$ is equal to the stable stage distribution ($\boldsymbol{w}_{1}$) (e.g. Kissel et al. 2014). Using eq. 7 in conjunction with the identity that $\boldsymbol{v}_{1}\boldsymbol{w}_{1}=\mathbf{I},$we then have:

$$\boldsymbol{n}_{1}=\boldsymbol{v}_{1}\boldsymbol{w}_{1}\boldsymbol{\lambda}_{1}^{1}\boldsymbol{w}_{1}$$

= $\mathbf{I}\boldsymbol{\lambda}_{1}^{1}\boldsymbol{w}_{1}$

$$={\boldsymbol{\lambda}_{1}^{1}\boldsymbol{w}}_{1}$$

$={\boldsymbol{\lambda}_{1}^{1}\boldsymbol{N}}_{0}$. (8)

Projection for an additional time unit gives:

$\boldsymbol{n}_{2}=\boldsymbol{v}_{1}\boldsymbol{\lambda}_{1}^{1}\boldsymbol{n}_{1}\boldsymbol{w}_{1}=\boldsymbol{v}_{1}{\boldsymbol{\lambda}_{1}^{1}\boldsymbol{\lambda}_{1}^{1}\boldsymbol{n}}_{0}\boldsymbol{w}_{1}=\mathbf{I}{\boldsymbol{\lambda}_{1}^{2}\boldsymbol{w}}_{1}=\boldsymbol{\lambda}_{1}^{2}\boldsymbol{n}_{0}$ (9)

Projecting from reintroduction ($T=c=0$) to the end of the managerial frame of interest, say, $T=T$, we have:

$\boldsymbol{n}_{T}=\boldsymbol{v}_{1}\boldsymbol{\lambda}_{1}^{1}\boldsymbol{n}_{T-1}\boldsymbol{w}_{1}=\boldsymbol{v}_{1}\boldsymbol{\lambda}_{1}^{T}\boldsymbol{n}_{0}\boldsymbol{w}_{1}=\mathbf{I}{\boldsymbol{\lambda}_{1}^{T}\boldsymbol{w}}_{1}={\boldsymbol{\lambda}_{1}^{T}\boldsymbol{w}}_{1}=\boldsymbol{\lambda}_{1}^{T}\boldsymbol{n}_{0}$. (10)

Rearranging, we have:

$\boldsymbol{n}_{0}=\frac{1}{\boldsymbol{\lambda}_{1}^{T}}\boldsymbol{n}_{T}$, (11)

which is the expression that specifies the abundances that are necessary in the instance of reintroduction ($T=c=0$) to achieve the predetermined future population target under stable dynamics by time $T$.

It is known that the stage or age distribution must be taken into account while determining a recovery threshold (Wiedenmann, Fujiwara, & Mangel 2009). Hence, given a predetermined number of breeding pairs at the target timeframe, we use the stable stage proportions to calculate the abundances in the non-breeding stages at that time. Suppose $a_{m}$ represents the predetermined number of breeding pairs in one stage that is desired at time$T$. Letting $\boldsymbol{n}_{T}$= $\left[ \begin{matrix} a_{1} & a_{2} & \ldots& a_{m} \end{matrix} \right]^{'}$ represent the vector of abundances of stages $1, 2, \ldots.m$ at time $T$, and the stable stage proportions (SSP) be $p_{1}=\left[ \begin{matrix} p_{11} & p_{12} & \ldots& p_{1m} \end{matrix} \right]^{'}$, then the total number of females in stage $m$ at time $T$ is:

$u=\frac{a_{m}}{p_{1m}}$. (12)

The remaining stage abundances at time $T$ are:

$\boldsymbol{n}_{T}=\left[ \begin{matrix} p_{11}u & p_{12}u & \ldots& p_{1m}u \end{matrix} \right]$. (13)

Applying the target abundance vector in eq. 13 to eq. 11 gives the stage abundances that must be introduced at the onset of time $T=0$to theoretically achieve the predetermined management goal using stable population theory by the end of time $T=T$.

Suppose $b$ represents the predetermined number of breeding pairs in a combined set of stages that are desired at time$T$. Let $\boldsymbol{n}_{T}$= $\left[ \begin{matrix} x_{1} & x_{2} & \ldots& x_{m} \end{matrix} \right]^{'}$ represent the vector of abundances of stages $1, 2, \ldots.m$ at time $T$, and the stable stage proportions (SSP) be $p_{1}=\left[ \begin{matrix} p_{11} & p_{12} & \ldots& p_{1m} \end{matrix} \right]^{'}$. Without loss of generality, let breeding pairs occur in stages $x_{m-1}$ and $x_{m}$. The desired number of breeding females at time $T$ must be equal to:

$b=x_{m-1}+x_{m}.$ (14)

The total number of females in stages $m-1$ and $m$ time $T$ is then:

$X=\frac{x_{m}+x_{m-1}}{(p_{1m}+p_{1m-1})}$. (15)

The stage abundances at time $T$ are then :

$\boldsymbol{n}_{T}=\left[ \begin{matrix} p_{11}X & p_{12}X & \ldots& p_{1m-1}X & p_{1m}X \end{matrix} \right]$. (16)

Applying the target abundance vector in eq. 16 to eq. 11 gives the stage abundances that must be introduced at the onset of time $T=0$to theoretically achieve the predetermined management goal using stable population theory by the end of time $T=T$.

LITERATURE CITED

Caswell H. 2001. *Matrix population models: Construction, analysis, and interpretation. 2^nd^ Ed.* Sinauer, Sunderland, Massachusetts, USA.

Fox GA & Gurevitch J. 2000. Population numbers count: tools for near-term demographic analysis. *American Naturalist* 156:242–256. DOI: 10.1086/303387

Kissel AM, Palen WJ, Govindarajulu P, & Bishop CA. 2014. Quantifying ecological life support: the biological efficacy of alternative supplementation strategies for imperiled amphibian populations. *Conservation Letters* 7(5):441-450. DOI: 10.1111/conl.12093

Wiedenmann J, Fujiwara M, & Mangel M. 2009. Transient population dynamics and viable stage or age distributions for effective conservation and recovery. *Biological Conservation* 142:2990-2996 DOI:10.1016/j.biocon.2009.07.031
