# Supplementary material for: How can we augment the few that remain? Using stable population dynamics to aid reintroduction planning of an iteroparous species
Source: PeerJ. 2019 May 2;7:e6873. doi: 10.7717/peerj.6873 (PMC6500717; doi:10.7717/peerj.6873)
Supplement: Supplemental Information 2 — The .txt files and R code that underlies the interactive software. [file peerj-07-6873-s002.zip › Finalized_Supplement_Software/Legend.docx]

Supplement Legend

Supplement to:

Hanley BJ, Bunting EM and Schuler KS. How can we augment the few that remain? Using stable population dynamics to aid reintroduction planning of an iteroparous species

This supplement contains the R software to generate the underlying text file, plus the R software for the interactive application. The R code and simulated data text files exist for 2-, 3-, 4-, and 5-stage life histories.
